# WHAT DO NURSING HOMES RECEIVING HIGH COMPLAINTS GOT TO DO WITH RESIDENTS’ QUALITY OF LIFE?

**DOI:** 10.1093/geroni/igad104.3694

**Published:** 2023-12-21

**Authors:** Jenny Kwon, Xiao Qiu

**Affiliations:** Miami University, Oxford, Ohio, United States; Miami University, Oxford, Ohio, United States

## Abstract

Residents’ quality of life is important to understand nursing home (NH) quality. Official complaints are another NH quality indicator. However, little research has examined the two quality measures together. To fill this gap, this study aims to investigate the association between resident satisfaction and NH complaints. 2018–2019 complaints data from the Ohio Department of Health, 2017 Ohio Biennial Survey of Long-Term Care Facilities, and 2017 Ohio Nursing Home Resident Satisfaction Survey were merged (N=629). Eight outcome variables were used: overall resident satisfaction score and seven individual domain scores. They range from 0 (low) to 100 (high). For independent variables, we labeled NHs received 6 or more complaints per 100 residents in both 2018 and 2019 as ‘high complaint repeaters.’ Multiple linear regression was conducted. There is a statistically significant relationship between official complaints and resident satisfaction. High complaint repeaters had lower overall satisfaction score by 1.48 (p < .01); lower moving in score by 2.16 (p < .01); lower spending time score by 1.30 (p < .05); lower caregivers score by 1.20 (p < .05); lower meals and dining score by 2.18 (p < .05); lower environment score by 1.13 (p < .05); and lower facility culture score by 2.14 (p < .01) than non-high complaint repeaters. However, there is no association between complaints and care and services domain score. State policy makers need to consistently monitor high complaint repeaters and support them to reduce complaints. Also, providers should allocate resources and design strategies to improve residents’ experiences.

